# Division of labour during honeybee colony defence: poetic and scientific views

**DOI:** 10.1098/rstb.2023.0272

**Published:** 2025-03-20

**Authors:** Daniela Ramirez-Moreno, C. Giovanni Galizia, Morgane Nouvian

**Affiliations:** ^1^ Department of Biology, University of Konstanz, Konstanz D-78457, Germany; ^2^ Centre for the Advanced Study of Collective Behaviour, University of Konstanz, Konstanz D-78457, Germany; ^3^ Zukunftskolleg, University of Konstanz, Konstanz D-78457, Germany

**Keywords:** honeybee, defence, soldier bees, guard bees

## Abstract

Poets, philosophers and politicians have used bees, and often projected an idealized human society into their view of how beehives are organized, from the ancient Greeks to present times. We first review how division of labour in honeybees was perceived by human observers, before presenting our current understanding. We focus specifically on defensive behaviour and show that this model provides an interesting case study for our conceptual understanding of division of labour as a whole. We distinguish three phases of the defensive response: detection of an intruder, recruitment of individuals into collective defence and attack. Individual bees may selectively contribute to one or more of these steps. Guard bees monitor entering conspecifics or attacking mammals, and release an alarm pheromone to recruit stinging soldiers. However, we are still far from understanding why only subsets of bees become guards or soldiers (or even if soldiering can be considered a task *per se*). We discuss the stimuli associated with each of these steps, how they define the number of bees needed and how they might combine with individual and developmental characteristics such that individuals take on a particular task. We also highlight pending questions and interesting avenues for future research.

This article is part of the theme issue ‘Division of labour as key driver of social evolution’.

## 1. Introduction

Diversity of behaviours within a species is ubiquitous in the animal kingdom, both within and between individuals. Within an individual means along the ontogeny of an animal. In the simplest case, early stages are more devoted to growth, while later stages are devoted to reproduction. In reproduction, different sexes fulfil different tasks, even at equal ages. By contrast, synchronous division of labour refers to when a given task could be fulfiled by different (or potentially all) individuals but is only accomplished by some. This is a more complex trait, and particularly interesting in social species. Here, the question of how the division is organized becomes relevant: how does the individual, in a social context, decide whether to fulfil a particular behaviour, and not another one? This decision is crucial for survival of both the individual and the social group: if all individuals in a group would select the same behaviour, or if some necessary behaviours are not selected at all, the group cannot function efficiently anymore.

Human societies have particularly complex systems for labour division, and these systems differ in different cultures and across human history. The underlying rules are socially dictated and maintained, and generally justified with some sort of philosophical or political rationale. For example, a king might claim that his power, and the organization in his kingdom, is dictated by divine intervention, and thus justified beyond human control. Historically, a comparison with non-human species to strengthen the authority of the argument has often been used. Social insects, including ants, bees, wasps and termites, yield themselves as perfect examples, and have been used as justification for division of labour in human societies since ancient times. Clearly, the most frequently used example among social insects is given by the honeybee, *Apis mellifera*, to show how complex and efficient a society can be, if well organized.

It should be noted that human thinkers have looked at division of labour in honeybees over the centuries generally using a normative attitude, rather than an investigative: how can we use bees to understand human society, and to derive rules for peaceful convivence? The Greek philosopher Aristotle (384–322 BCE) saw the bees as ‘zoon politicon’, as a political and social animal, a role that he also attributed to humans: the key element here was the *polis*, i.e. the city/state, which both bees and humans had in common [1]. The Latin poet Virgil (70–19 BCE) saw the bee state as a perfect society: sex-free, and divinely ordered—quite in contrast to his contemporary Roman society that was trapped in civil wars and political assassinations. Describing the bees, he writes: ‘the leader is the guardian of their labours: to the leader they do reverence, and all sit round the leader in a noisy throng, and crowd round in large numbers, and often they lift the leader on their shoulders and expose their bodies in war, and among wounds, seek a glorious death’ (*Georgics*, book IV, lines 214−218). Virgil describes hive defence emphatically as defence of the bee king. In the play *Henry V*, William Shakespeare (1564−1616 CE) lets the Archbishop of Canterbury dictate the good rules of society, insisting on the fact that different groups have different tasks:

therefore doth heaven divide the state of man in diverse functions, setting endeavour in continual motion; to which is fixed, as an aim or butt, obedience: *for so work the honey-bees*, creatures that, by a rule in nature, teach the art of order to a peopled kingdom. They have a king, and officers of sorts: where some, like magistrates, correct at home; others, like merchants, venture trade abroad; others, like soldiers, armed in their stings, make boot upon the summer’s velvet buds; which pillage they with merry march bring home to the tent-royal of their emperor: who, busied in his majesty, surveys the singing masons building roofs of gold; the civil citizens kneading-up the honey; the poor mechanic porters crowding in their heavy burdens at his narrow gate; the sad-eyed justice, with his surly hum, delivering over to executors pale the lazy yawning drone. [2] (emphasis added)

Note the complexity of the bee society described by Shakespeare, and still the centrality of the king’s role!

In 1586 Luis Mendez de Torres observed that the 'king’ in fact was a queen, and Charles Butler (1571−1647) placed the resulting political implication into the title of his book about beekeeping: ‘The feminine monarchy’ (1609). Jan Swammerdam (1637−1680) found the ovaries in his microscopical studies, and thus confirmed that the queen was female. Johan Jakob Bachofen (1815−1887) did not wait long: in ‘Das Mutterrecht’, he describes a putative prehistorical development of human society from matriarchy (he does not use that term) to patriarchy, and uses the bee to strengthen the notion of how matriarchy may be natural for a primitive society [3,4]. In summary, bees have been used over centuries as normative examples for how a human society may need to look—though, often the view of the beehive was more a mirror of the human society, rather than the other way round. It is no surprise, then, that the current view of the organization in the beehive does not see the queen as a ruler, but rather the workers, who control the actions in the hive via pheromones and collective behaviour: the ‘honeybee democracy’ [5] appears now as a mirror of current societal thinking.

A fundamental question in honeybee collective behaviour is: how does the individual bee decide which task to fulfil, such that the colony functions properly? That is: given the many different tasks to be accomplished in the hive (nursing, defending, foraging, etc.), and the large number of individuals involved (up to 60 000 bees in a single hive), how are tasks allocated? Some are genetically and epigenetically controlled: the males, the queen and the workers. Within the (female) workers, bees use age polyethism: young bees are nurses, old bees are foragers (to name the two major tasks), but also cleaning, comb building, nest defence, humidity and temperature control etc., need to be accomplished. There is plenty of plasticity in the transition phases, regulated by pheromones and hormones such as the juvenile hormone (JH), in order to ensure that enough individuals are allocated to each task at all times [5]. Studies of division of labour in honeybees and in social insects more generally have mainly focused on the two major tasks of nursing and foraging, and developed models suited to these tasks. We’ll focus this review on colony defence against large predators, and argue that this task offers a different perspective because of its episodic nature and riskiness. To structure our discussion, we’ll divide colony defence into three steps: detection of the threat, recruitment of additional defenders and attack of the intruder.

## 2. Threat detection

**Figure 1.**
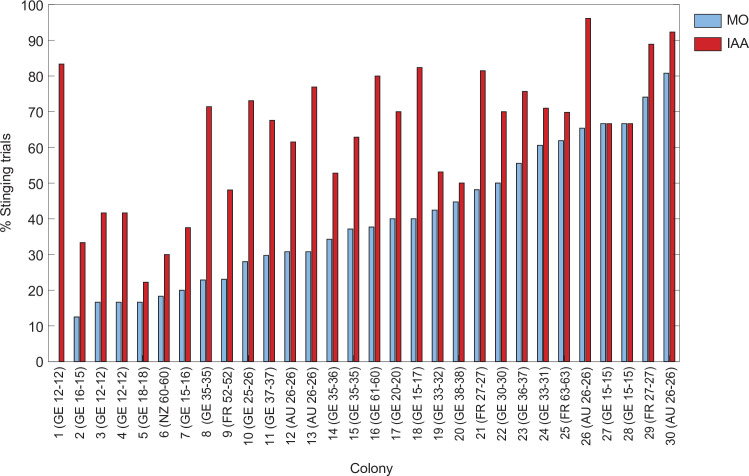
Stinging responsiveness to a rotating dummy alone (solvent control with mineral oil (MO), in blue) or to the same rotating dummy in the presence of alarm pheromone (10% vol/vol IAA in mineral oil, in red) for bees taken from 30 different colonies in 4 different countries (AU: Australia; FR: France; GE: Germany; NZ: New Zealand). The bees were tested once in a small arena, in pairs, as described in [8]. A stinging trial corresponds to at least one of the bees stinging the dummy. The numbers in parentheses indicate the number of bee pairs tested with MO and IAA, respectively. The colonies are ranked according to their response to the MO control. IAA generally increases stinging responses, but not always.

The success of hive defence relies on the ability of honeybees to detect threats and to respond appropriately [6,7]. This is a crucial step because it is gating the defensive response as a whole. In figure 1, we compiled the data of 30 colonies for which we tested the stinging response of pairs of bees in a standardized assay (described in [8]). Looking at the responsiveness to the visuo-tactile stimulus alone (rotating dummy, control situation with mineral oil, in blue), the variability is enormous: it spans the whole range from 0 to 81% of the pairs stinging depending on the colony. Similarly, cross-fostering experiments using field tests found that colony defensiveness was mainly correlated with the likelihood of a particular genotype to initiate stinging [9].

Threat detection appears to be the task of a subset of bees, called guards. They detect potential enemies such as bees from other hives, other insects and larger predators like humans through olfactory, visual or mechanical cues [10–13]. Guards are located at the hive entrance and stand with their forelegs off the ground and their antennae pointing forward ready to inspect landing bees [13,14]. Their primary task is to patrol the entrance to identify and reject non-nestmates by mauling them [14]. Guarding is a brief occupation performed by a small group of middle-aged bees (approx. 15% of bees ever become guards) [13]. The proportion of guards is variable [15] and positively correlated with the stinging response [16]. Although guarding is a specialized task, it does not seem to require prior learning or morphological differentiation [13]. What then makes a bee a guard bee?

Honeybees’ division of labour is based on temporal polyethism, where tasks are allocated according to bees’ age [17,18]. While guards do not differ morphologically from other bees, their physiological development could set them apart. Guards transition to foraging earlier than most other bees, suggesting that their developmental rate is slightly faster [19]. Indeed, guards also have higher levels of JH than other bees of the same age, similar to those of older bees [20]. JH levels show more variability in middle-aged bees, presumably due to the different tasks performed by bees at that age [21]. Elevated JH levels may be linked to increased metabolic activity and to some extent to positive phototaxis [20–22]. Since the JH levels of guards and foragers are similar [20], additional physiological differences must be involved. Detection and discrimination of threats by guards involves specific visual and olfactory cues, that guards may be more attuned to than other bees.

Vision is fundamental for detecting threats during guarding. Guards are able to recognize the typical flight of robber bees [14] and the proximity of other unfriendly visitors. Guards often fly up to inspect disturbances around the hive, but do not intercept robbers in the air [13]. The relationship between age polyethism and visual maturation is not well understood yet, but we know that the visual system changes over time [23]. Do guards have special visual capacities? In the case of the eusocial stingless bee *Tetragonisca angustula*, a transient increase in the relative volume of the optic lobes, the brain structures involved in visual processing, is observed in hovering guards [24,25]. Whether honeybee guards would undergo similar brain plasticity is unknown. Guards tend to be more active during the day [26]; therefore, it seems guarding is under circadian regulation that might correlate with foraging activity and the probability of being robbed. As they transition from indoor tasks to foraging, bees gradually become more attracted to light [27] and this goes hand in hand with the development of the visual system [28]. The link between phototaxis and guarding has not been directly addressed. However, it is possible that guards would exhibit a stronger preference for light than other middle-aged bees, considering that guarding occurs at the hive entrance. Exposure to light leads to a reorganization in the visual part of the mushroom bodies (the collar), increases JH titres [21] and increases the levels of opsin and arrestin mRNAs [29]. These mechanisms might reinforce small differences between middle-aged bees. The potential impact of these rearrangements on the current or subsequent life stages of guards remains uncertain.

In addition to visual cues, guards discriminate between nestmates and non-nestmates with high efficiency using olfactory cues [10,30–32]. Guards know the odour of their colony and use it as a template to accept or reject bees [10,31,33]. This signature mixture comprises both environmental and synthetized compounds [12,34]. Intruders are more likely to be rejected if they are experimentally placed at the hive entrance than if they are placed directly inside the colony, even after contacting around 30 bees [35]. This means that guards, at the entrance, are either more attentive or more sensitive to olfactory cues than an average bee. They also respond to olfactory cues not only from other bees [31] but from humans and animals [36]. These discrimination abilities—visual and olfactory—are either not present in other bees, or are not followed by defensive behaviour [30].

The leading model to explain how division of labour arises is the response threshold model, which postulates that different individuals have varying thresholds for task-related cues. Individuals with low thresholds for a particular cue would tend to take over the related task, thereby decreasing the cue and preventing individuals with higher thresholds from engaging in this particular task—thus remaining available for other tasks [37]. What could be a task-related stimulus for guarding? An interesting conundrum of the task of guards is that their daily role seems to be defence against non-nestmate, for which the cues are the characteristic flight pattern of robber bees and cuticular hydrocarbon profiles. These are very different from the cues given away by large predators, yet guards seem to be in charge of detecting these threats as well. This suggests that either guards are better at visual/olfactory discrimination in general, as we discussed above, or they are more attentive to such sensory information. Guards may also be more sensitive to other types of stimuli, for example, it would be interesting to evaluate their responsiveness to mechanical stimuli, which are also known to trigger defensive responses [38]. Bees of guarding age responded by stinging to lower voltages than other bees [39], suggesting that they may be more sensitive to noxious stimuli. However, the opposite was found when comparing the first responders to a disturbance (taken to be guards) and foragers [40].

The inter-individual variability necessary for division of labour to develop according to a response-threshold model could be created by genetic factors. Indeed, genotype influences the bee’s propensity to guard [9,34,41,42]. Three quantitative trait loci (QTLs) related to guarding and stinging have been identified: *Sting 1*, *Sting 2* and *Sting 3* [43]. Stronger defensiveness is linked to African ancestry (AHB; *scutellata*-hybrid) compared to European races (EHB) [44,45]. Unmanipulated AHB colonies tend to have more guards, and these individuals guard for longer times than EHB guards [9]. In mixed colonies, bees of Africanized descent were more likely to initiate guarding than co-fostered European bees [46]. AHB guards also seem to have lower thresholds to initiate an attack [9]. All these traits likely contribute to the overall greater defensiveness of AHB.

On top of these genetic determinants, guarding behaviour is affected by a number of environmental factors. In periods of nectar dearth, bees tend to rob each other more often [47,48]. Both robbed and robbing colonies react by increasing the number of guards and the individual guarding effort [35,49]. When robbing pressure decreases, the number of guards follows [47]. This could be attributed to an improvement in conditions resulting in less motivation to attack or/and the availability of new tasks to perform like food processing, detracting bees from guarding. The presence of empty combs in the colony results in faster defensive responses to a visual target but not to the alarm pheromone [50]. This could presumably be mediated by increased guarding efforts, although this study did not evaluate the role of guards specifically. According to the response threshold model, additional guards (or replacements of killed guards) would take over this task because they encounter more ‘guarding-related stimuli’, for example, they come in contact with more non-nestmates inside the colony. If guards have intrinsically different discrimination abilities, one would expect continuous removal of guards to result in new guards being less and less efficient over time, as the pool of ‘good discriminators’ dwindles. It would be interesting to test this hypothesis. Whether guarding behaviour is also affected by repeated disturbances from vertebrate attacks over long time scales has not, to our knowledge, been tested. This would be another interesting experiment to do, considering that the stimuli involved would be episodic in nature and therefore not easily reconciled with a response-threshold model. Indeed in this model, a persistent guarding-related stimulus would be necessary for the number of guards to remain high also in between attacks. It is not clear what such stimulus could be when the predator is absent. Finally, further exploration is needed to understand the extent of tasks performed by guards, such as their potential involvement in other activities like foraging [14,15,51] and whether bees that guard exhibit an increased inclination towards aggressive behaviour later in life (i.e. become soldiers).

## 3. Recruitment

Honeybee colonies attract many predators that are too large for a few guard bees to handle. Hence, the second crucial element of the defensive response is recruitment. Thanks to this process, honeybees can convert the alert given by a small number of guard bees into an attack by hundreds of soldiers. The recruiting step thus serves to couple two tasks that necessitate very different numbers of workers: optimal vigilance might be already achieved by a small workforce, but actually deterring the predator requires many more defenders. Recruitment can take several forms. Acoustic responses during disturbances such as hissing or piping have been reported, but it is unclear if they are used to alert other bees, to threaten the predator or both [52–55]. In *A. cerana*, defenders are recruited visually to perform an ‘I see you’ signal that deters hornets [56,57]. The open nester *A. dorsata* also responds to hornets with a similar behaviour termed shimmering [58]. Recruitment, in this case, is linked to proximity with aroused bees [59] and may occur through direct mechano-sensation, nest vibrations [60] and/or Nasanov pheromone release [61]. Whether such signalling pathways involve task specialization of either the emitter or the receiver bees is not known, although there is some evidence that shimmering is started by bees at a specific location on the nest [62].

**Figure 2.**
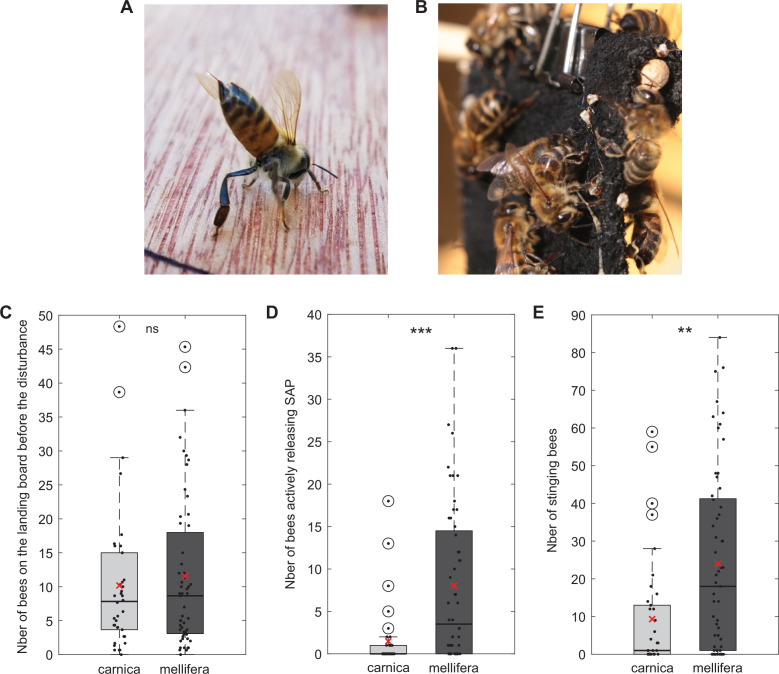
(A) Bee actively releasing the sting alarm pheromone (SAP). Credit: Jessica Helgen, UMN Bee Squad. (B) Stinging bees and embedded stingers on a black suede flag. Credit: David Vogel. (C) Prior to the disturbance, the number of bees on the landing board of the colonies tested were similar for the two subspecies *carnica* and *mellifera*. This number was obtained by averaging the number of bees visible at 3 time points: 2 min, 1 min and 1 s before the test. Mann–Whitney *U*-test, *U* = 1734, *z* = −0.403, *p* = 0.687. (D) More individuals from *A. m. mellifera* colonies were observed actively releasing the SAP during the disturbance, which consisted of a black suede flag moving up and down in front of the colony entrance. This test lasted 2 min. The flag was initially placed 4 cm away from the entrance and was moved to 12 cm after 1 min. Mann–Whitney *U*-test, *U* = 1276, *z* = −4.272, *p* < 0.001. (E) *A. m. mellifera* colonies developed a much stronger defensive response, as evidenced by the higher number of stingers embedded in the flag after 2 min. Mann–Whitney *U*-test, *U* = 1410, *z* = −3.189, *p* < 0.01.

In Western honeybees, recruitment mainly operates via the release of alarm pheromones. The sting alarm pheromone (SAP) is produced by the stinger and stored on a hairy membrane surrounding the base of the sting shaft, allowing for a fast release whenever the stinger is exposed (see [38] and references therein). Bees can actively disperse the SAP by raising their abdomen, extruding their stinger and fanning their wings (figure 2A). They often do so while running into the colony, where other bees may not be able to perceive the threat directly [63]. This active release of the SAP has received little attention past its original description. In a preliminary experiment, we tested five colonies of *Apis mellifera carnica* and seven of *Apis mellifera mellifera* for their defensive behaviour. We found that *A. m. mellifera* exhibits this behaviour much more frequently than *A. m. carnica* (figure 2C,D). This may in turn explain their greater overall stinging responses (figure 2E) since this initial step contributes to determining the number of bees recruited. It would be interesting to study this behaviour in more detail, in particular whether it is part of the guards’ repertoire or whether it is a separate component of the defensive response with its own molecular underpinnings and regulation. Active SAP release is specific to large intruders that require many stinging defenders, it was never reported in response to conspecifics or wasps.

Over 40 compounds make up the SAP [64–67], a number in stark contrast with the single to few compounds that are reported in most known alarm pheromones [68,69]. Only a few of these compounds may be behaviourally active, but an —as yet unproven—alternative hypothesis would be that this diversity serves to modulate recruitment. The components might spatially segregate based on their volatility and thus create different zones around the point of release, each triggering a specific behavioural response (‘active space’ concept, reviewed in [70]). Indeed, Wager & Breed [71] tested 11 SAP compounds and found that each elicited only part of the behavioural sequence (exiting the colony, flying, locating the target). Some compounds are also known to prolong the activity of the main component, isoamyl acetate (IAA) [66,72], thus creating a temporal pattern: highly volatile compounds quickly trigger recruitment, while less volatile compounds maintain the signal.

Both the quantity and the composition of the SAP vary across bees, which may further determine how recruitment is shaped at the emitter’s end. Highly aggressive Africanized bees produce many compounds in higher quantities, and the defensiveness of colonies is correlated with their production level for these compounds [73]. They also produce an additional compound that synergizes with the action of IAA [44]. Within members of a colony, SAP production remains low until the bees reach the age at which they start guarding or foraging, around 20 days [74,75]. This means that young bees would not be able to efficiently recruit other bees during an attack (if they participated). Even among older bees, some compounds are only produced in high quantities by a small fraction (4%) of bees [74]. It is noteworthy that 2 of these, benzyl acetate and 2-nonanol, were included in the functional screen by Wager and Breed and their action also deviated from that of the other compounds: they both reduced flight activity instead of increasing it, and 2-nonanol repelled bees in a Y-maze assay [71]. In the Eastern honeybee *Apis cerana*, benzyl acetate is produced in greater proportion by foragers and seems to repel bees from feeders [76]. Is this also the case in *A. mellifera*? This would raise a number of other questions, such as: are foragers involved in recruitment during colony defence, or do they only communicate danger at foraging sites? If they do release the SAP in the nest, how does the potential presence of this additional compound affect the reaction of nearby bees (compared to the one elicited by SAP release from guard bees)? More generally, do variations in SAP composition contribute to division of labour during defence?

Division of labour may also arise at the other end, from variations in how receiver bees perceive or react to the SAP. Peripheral detection of SAP compounds is stable after 6 days, according to electro-antennograms [74]. However, there is no data available regarding alarm pheromone processing in the central nervous system of honeybees of different ages or different tasks. In the stingless bee *T. angustula*, the peripheral olfactory response to citral, a defence-related odour, is higher in hovering guards than in foragers [77], lending support to the idea that this could vary also among honeybees. Responsiveness to the alarm pheromone was assayed using groups of caged bees, which reacted to IAA by flickering their wings and increasing locomotion [78,79]. According to these studies, SAP responsiveness increases with age, and this pattern can be accelerated by treating the bees with an analogue of the JH. However, these results are somewhat limited in that the behavioural response that would be triggered in a more naturalistic context is not clear.

The act of stinging also exposes the setaceous membrane and thus automatically releases the alarm pheromone. The elastic skin of vertebrates traps the barbed stinger of honeybees (figure 2B), which then detach from the abdomen—a process called stinger autotomy [80]. The lone stinger continues to pump venom into the wound but also to release the alarm pheromone, effectively tagging the intruder. This creates a positive feedback loop, attracting even more recruits to sting this particular target [81]. As a result, the SAP is released dynamically during an attack, not only at the beginning. This variation in SAP concentration is meaningful, and shapes the collective output. Indeed, bees become more likely to sting as the SAP concentration increases, but only up to a certain concentration. At even higher levels, the likelihood to sting decreases [82]. Using modelling, this study suggested that starting low is useful to quickly disengage in case of false alarms, and that the plateau extends until a concentration corresponding to the number of stingers required to deter the most resilient predators. The subsequent decrease in stinging likelihood at high concentrations may be due to a lack of adaptation (no response could evolve because these concentrations were never encountered) or to an inhibiting mechanism that preserves the workforce [82]. This responsiveness pattern may also contribute to distribute stingers across predators or body parts, if they are sufficiently distant to be recognized as carrying different concentrations of alarm pheromone [81]. This model, however, did not take into account division of labour. Does SAP responsiveness vary across bee types? And if so, does that create selective recruitment of some populations at different times of the defensive response? These questions merit further attention.

Bees can thus perform 2 distinct actions to initiate the defensive response: one is the active release of alarm pheromone that we described above, the other is stinging the intruder. It is important to note that the consequences of these actions are quite different. In terms of recruitment, releasing the alarm pheromone inside the hive or at its entrance, where many potential recruits are located, is likely the most efficient. However, this chain reaction may delay the stinging response, to the advantage of the predator. The difference is even more dramatic from the bee’s point of view: running into the colony to disperse the alarm pheromone effectively means running to safety, while stinging a vertebrate inevitably ends with the death of the bee. According to a study by Arechavaleta-Velasco & Hunt [34], less than 3% of guards actually stung during a simulated attack. Nonetheless, the proportion of guards that stung correlated with the overall number of stingers and removing guards prior to the disturbance decreased the number of stinging bees. If guards don’t sting, it would be tempting to assume that their role is to recruit nestmates via active alarm pheromone release. But these authors did not observe such behaviour: they only report that guards were ‘extremely agitated and active’, and conclude that their role was ‘not clear’ [34]. Thus, more work is needed to determine the exact role played by guards during vertebrate attacks.

## 4. Attack

After detecting a potential threat, bees first need to decide whether to attack or retreat [83]. The attack itself strongly depends on the nature of the unfriendly visitor: hornets are engulfed in heating balls, ants are blown away by wind generated by fanning the wings, mammals are stung [38]. Here, we will focus on collective stinging responses, which are believed to have evolved in response to vertebrate predation [80,84–86]. This type of attack has shaped honeybee–human interactions through history, even being used as a war strategy to deter adversaries [87]. As of today, bee fences are still used to protect crops and trees from elephants [88]. Although we focus on stinging as the most efficient deterrent, it is not the only behaviour exhibited by bees during collective defence: they also fly, buzz, head-butt, bite and pull the hairs of intruders—until it becomes so unpleasant for the attacker that it is forced to retreat [89]. How individuals decide which of these responses to adopt, or whether different subsets of workers are involved, has not been studied. Interestingly, bees continue intimidating and harassing the intruder after stinging (and hence losing their stinger), thus preserving some value as defenders [90].

Bees that either sting or pursue the predator in response to the alarm pheromone are known as soldiers. Between colonies, the proportion of bees reacting to the alarm pheromone by stinging (figure 1; difference between the solvent control in blue and IAA in red) is strikingly variable: in some colonies, recruitment is barely visible (e.g. colonies 5, 20, 27, 28) whereas in others the number of stinging pairs was more than doubled in the presence of IAA (colonies 2−4, 8−13, 16, 18). Colony 1 even went from 0 to 83% of pairs stinging when the alarm pheromone was added. It would be difficult to predict from these data which colony would score the most aggressive during a field test, because different strategies (by which we mean different investments in detection versus recruitment/attack) may produce the same output.

The main question about soldier bees is: do they constitute a separate pool of bees, or do they derive from bees abandoning other tasks when the need arises? Unlike many other social insects, honeybee soldiers are not morphologically specialized for the task. Soldiers have less wing wear than foragers, suggesting that they spend more time inside the colony where they can be quickly recruited; and they are genetically distinct from guards and foragers, supporting the idea that colony defence can be subdivided into two tasks [91]. Nonetheless, Guzman-Novoa *et al*. [9] reported that 25% of stinging bees were previously marked as guards in field tests with Africanized colonies.

Whether soldiers are a separate taskforce might also depend on the colony considered. Most colonies seem to trade-off between foraging and defence [92,93], suggesting that some bees have to switch from foraging to attacking during defensive events. On the other hand, some (but not all) colonies of African descent, *A. m. scutellata* and *A. m. capensis*, can employ flying soldiers without compromising their foraging level [93]. Their soldiers were presumably not involved in foraging, which might indicate that they are fully specialized in defence. This difference in defensive investment may be linked to the exact environment and constraints that (sub-)species have to face. In the tropics, food is always available thus foraging is less limiting, or in other words: colonies can afford to have less foragers and more soldiers [94]. An alternative interpretation of this data would be that most colonies actively decrease their foraging rate as part of their defensive response. For example, Asian honeybees (*A. cerana*) produce a stop signal that inhibits departures from the colony in the presence of hornets [95]. More information is needed to disentangle these different hypotheses and conclude on the specialization of honeybee soldiers.

If soldiers are a specialized task force, then some factors must set them apart for this particular behavioural pathway. The age of soldiers range from 7 days old to the oldest bees (35 days old in this study [91]), which is similar to the age range of foragers but with an earlier onset (the youngest foragers observed in the same colonies were 13 days old). This challenges the idea that soldiers are old, dispensable bees; instead, it may be that soldiers are any bee with low value for the colony. Bees infected with *Nosema ceranae* engage in riskier tasks like robbing [96]. Viral infection with the Kakugo virus also seems to alter the bees’ behaviour, promoting aggression [97]. How health status relates with the propensity to attack and with the allocation of soldiers remains unclear and raises the question whether bees have a sense of their own value to the colony.

Soldiers are only involved in collective defence against large predators, which does not happen regularly (if it does, the colony may respond by absconding, i.e. by moving the entire nest to a safer location). In keeping with a response threshold model, what could be a continuous ‘soldier-related stimulus’? One idea would be that such stimulus is produced by the bees’ themselves, for example in the form of a pheromone. This could explain why cross-fostering experiments found that the genetics of the host colony is dominant over the individual’s [9,39]. Similarly, stinging responses are better correlated with the genetic structure of the colony as a whole than that of individual soldiers [98], which again points to a collective regulation of stinging behaviour. Another way that the colony could retain a pool of workers available for defence, would be that these bees have high thresholds for all other tasks. As of now, there is no data to either support or discard this hypothesis. Finally, the allocation of workers to the task of soldiering may rely on a different mechanism, yet to be identified. If soldiers are rather a flexible workforce, then their main feature is likely a high responsiveness to the alarm pheromone. With this evolutionary strategy, soldiers would only be mobilized when the colony is under attack. Some support for this hypothesis comes from the observation that the molecular mechanisms linked to aggression in bees are similar whether this behaviour is inherited or triggered by the alarm pheromone [99,100]. Empirical evidence challenging the response threshold model is emerging [101], as well as alternative models of division of labour [37,102]. Honeybee soldiers may represent an interesting case study for this field of research.

**Figure 3.**
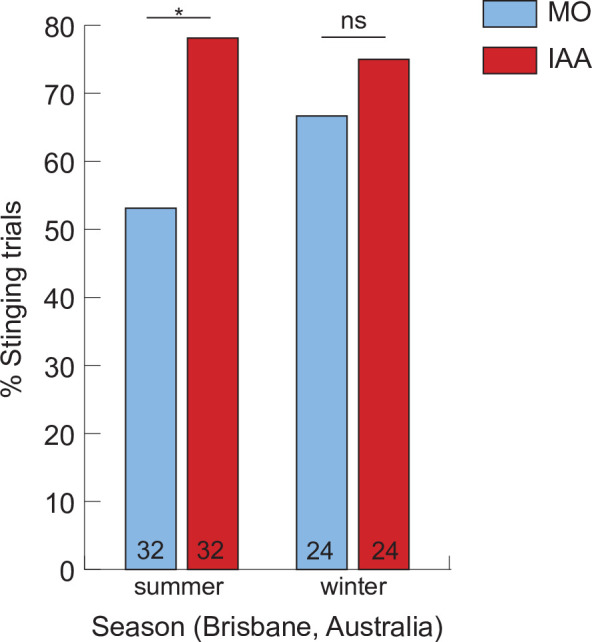
Stinging responsiveness of bees from the same 4 colonies at two different times of the year in Brisbane, Australia. The bees were faced with a rotating dummy in a small arena, in pairs, with or without the alarm pheromone (IAA) as already described for figure 1. Winter in Brisbane (June–August) is mild thus the colonies never stop foraging completely, but available resources are scarce. In winter condition, IAA did not significantly increase stinging propensity. Fisher exact tests, *p < 0.05, ns: not significant. Sample sizes are indicated inside each bar.

As a final level of complexity, the number of soldiers is dependent on environmental conditions [103]. Contrary to summer bees, winter bees do not respond to IAA by stinging [104]. This effect is visible as soon as the colonies start reducing their size in autumn, despite high activity levels and apparently ‘normal’ division of labour (figure 3 in Australia; M.N., personal observation, in late summer in Germany). This may imply that colonies are not able to allocate workers to the soldier task when resources become scarce. Notably, more guards are present at the hive entrance in such times [47] and colonies with more empty combs—as could be expected as a result of food shortage—tend to be more responsive to visual stimuli, but not to the alarm pheromone [50]. This trend is also visible in our data (figure 3), albeit not significant. Thus, it may be that task allocation is shifted from soldiers to guards in such circumstances. The adaptive significance of such shift remains to be investigated.

## 5. Conclusions

Honeybees have been used for centuries as a model society, highly organized and efficient—although the perceived hierarchy in this society was more a reflection of current political ideas than actual biology. Division of labour is at the core of this perceived efficiency and relies on individual specialization as well as communication within and between taskforces, such that an appropriate collective output is generated. Honeybee nest defence is a good example of such organization: it can be described by three core steps - detection, recruitment and attack - which may be performed by 2 subsets of bees—guards and soldiers—communicating via the sting alarm pheromone. This apparently straightforward structure, however, does not hold to scrutiny: comparing colonies or subspecies reveals interesting variations around this theme, and it is still unclear if soldiers can even be considered a separate pool of workers. Moreover, many questions remain unanswered regarding the mechanisms that may distribute bees between these tasks, especially taking in consideration that this allocation is very sensitive to environmental conditions. We suggest that answering these questions could significantly improve our conceptual understanding of division of labour.

## Data Availability

A small amount of new data was integrated in this review. It is available in Dryad [105].
